# SCD5 restored expression favors differentiation and epithelial-mesenchymal reversion in advanced melanoma

**DOI:** 10.18632/oncotarget.24085

**Published:** 2018-01-09

**Authors:** Rossella Puglisi, Maria Bellenghi, Giada Pontecorvi, Alessandro Gulino, Marina Petrini, Federica Felicetti, Lisabianca Bottero, Gianfranco Mattia, Alessandra Carè

**Affiliations:** ^1^ Center for Gender-Specific Medicine, Oncology Unit-Istituto Superiore di Sanita’, Rome, Italy; ^2^ Department of Oncology and Molecular Medicine, Istituto Superiore di Sanita’, Rome, Italy; ^3^ Department of Health Science, Tumor Immunology Unit, Human Pathology Section, Palermo University School of Medicine, Palermo, Italy

**Keywords:** melanoma, SCD5, miR-221&222, differentiation, mesenchymal-to-epithelial transition

## Abstract

Our previous data supported a role for the Stearoyl-CoA desaturase (SCD5) in protection against malignancy, whereby it appears to functionally modify tumor stroma impairing tumor spread. SCD5 is significantly expressed in primary melanoma, but becomes barely detectable at tumor advanced stages. Looking for the regulatory mechanisms underlying SCD5 reduced expression during melanoma progression, we demonstrated a significantly lower stability of SCD5 protein as well as the direct targeting of SCD5 mRNA by the oncogenic miR-221&222 in metastatic cell lines. Moreover, our results indicated the existence of a negative feedback loop between SCD5 and miR-221&222, in good agreement with their opposite functions. Also, we showed how SCD5 re-expression and the direct supplementation of its main product oleic acid (OA) can drive advanced melanoma cell lines toward differentiation and reversion of the epithelial-mesenchymal (EMT)-like process, eventually inducing a less malignant phenotype. Indeed, SCD5 re-established the sensitivity to all-trans retinoic acid in A375M metastatic melanoma, associated with increased levels of Tyrosinase, melanin production and reduced proliferation. As evidenced by the correct modulation of some key transcription factors, SCD5 managed by favoring a partial mesenchymal-to-epithelial (MET) transition in *in vitro* studies. Interestingly, a more complete MET, including E-cadherin re-expression correctly localized at cell membranes, was obtained in *in vivo* xenograft models, thus indicating the requirement of direct contacts between tumor cells and the surrounding microenvironment as well as the presence of some essential factors for SCD5 complete function.

## INTRODUCTION

Lipid composition affects membrane functionality, endosomal trafficking and provides a platform for cell signaling. Perturbation of this composition can have profound effects in different cell systems, including cancer cells [1]. Fatty acids are characterized by different chain length, linkage and saturation level, the latter characteristic influencing cell malignancy. Saturated fatty acids (SFA) (mostly 16:0 palmitic and 18:0 stearic acids) are desaturated by Stearoyl CoA desaturases (SCDs), SCD1 and SCD5 in humans. The action of these enzymes produce monounsaturated fatty acids (MUFA), essentially 16:1 palmitoleic and 18:1 oleic fatty acids, playing a role in the increment of cell membrane fluidity, but more important in the maintenance of the growth rate of tumor cells [2–4]. Different studies suggest a more complex picture on fatty acid role when we look at cancer cell ability to metastasize. For example in human oral carcinomas, the cell population able to metastasize express high levels of CD36 fatty acid receptor and diets rich in saturated palmitic acid increase the metastatic potential of this cell subpopulation [5]. On the contrary, in breast cancer cells with Her-2/neu oncogene amplification, the MUFA oleic acid suppresses Her-2/neu overexpression, which, in turn, synergistically interacts with anti-Her-2/neu immunotherapy by promoting apoptotic cell death [6].

SCD1, the main known human desaturase, is up-regulated in the majority of cancers and it is one of the central targets of growth factors and hormones that regulate key cell cycle events [7]. Differently from SCD1, the pathophysiological role of SCD5 remains basically unknown. Although the mechanism of action on lipid substrates appears the same, tissues and levels of expression are different. If SCD1 is ubiquitously present, SCD5 is essentially expressed in pancreas and in the central nervous system [4, 8]. During melanoma progression SCD5 expression was down-regulated, being highly expressed in primary compared to more advanced melanomas, where it is barely detectable. According to SCD5 antimetastatic function, its restoration reduces the capability to disseminate of both the A375M human melanoma and 4T1 murine mammary carcinoma cell lines evaluated in *in vivo* models. The reduced malignancy of SCD5 expressing cells was triggered by increased level of oleic acid, intracellular pH reduction with consequent failure of both vesicle movement toward the cell periphery and release of protumoral proteins, as Secreted Protein Acidic and Rich in Cysteine (SPARC) and Collagen IV [9, 10].

SPARC expression has been linked with aggressive, mesenchymal-like phenotypes in different human cancers, including melanoma, where it is known to contribute to phenotype changes during the Epithelial-Mesenchymal Transition (EMT) process when cells, losing their characteristics, gain mesenchymal features, become motile and eventually increase their dissemination capability [11]. The EMT event is regulated by different transcription factors and a hallmark of this functional change is the lack of E-cadherin expression. Literature data describe in depth the key proteins involved in the progression of epithelial tumors, but the functions of these factors are less clearly elucidated in non-epithelial contexts, like melanoma. It is also important to consider the general reversibility of this process.

A pivotal role has recently been provided for the Microphthalmia-associated transcription factor (MITF), master regulator of melanocyte differentiation and pigmentation genes [12], also involved in the control of different plasticity states. Several other genes, beyond MITF, can be associated with cell modification and dissemination [13], including SPARC, whose suppression reduces the tumorigenic potential in mouse xenograft assays [14–16].

In the present study we searched for the regulatory mechanisms and factors underlying SCD5 expression in differently staged melanoma cell lines. Also, in view of our previous studies demonstrating the SCD5-dependent intracellular retention of SPARC and considering the known involvement of SPARC itself in supporting a mesenchymal-like phenotype, we looked for the possible SCD5 capability in reversing the EMT-like process in melanoma. Actually, SCD5 re-expression in advanced melanomas was able to favor a less-invasive, more differentiated phenotype associated with a partial remodulation of the EMT. Interestingly this reversion appeared more complete in xenograft models, thus addressing the action of some essential constituents available and/or activated only *in vivo.*

## RESULTS

### Regulation of SCD5 expression in melanoma

Our previous studies showed SCD5 significantly higher expression in primary than in metastatic melanoma cell lines where it was barely detectable. Immunohistochemistry analysis performed on human melanoma bioptic specimens confirmed the strong positivity in primary cutaneous melanoma and the faint signal in metastatic samples (Figure 2B and ref [9]). More important, the enforced expression of SCD5 in the A375M metastatic melanoma cell line was able to significantly reduce its aggressiveness including the capability of these cells to produce metastases in an *in vivo* model of Nu/Nu mice [9].

**Figure 1:**
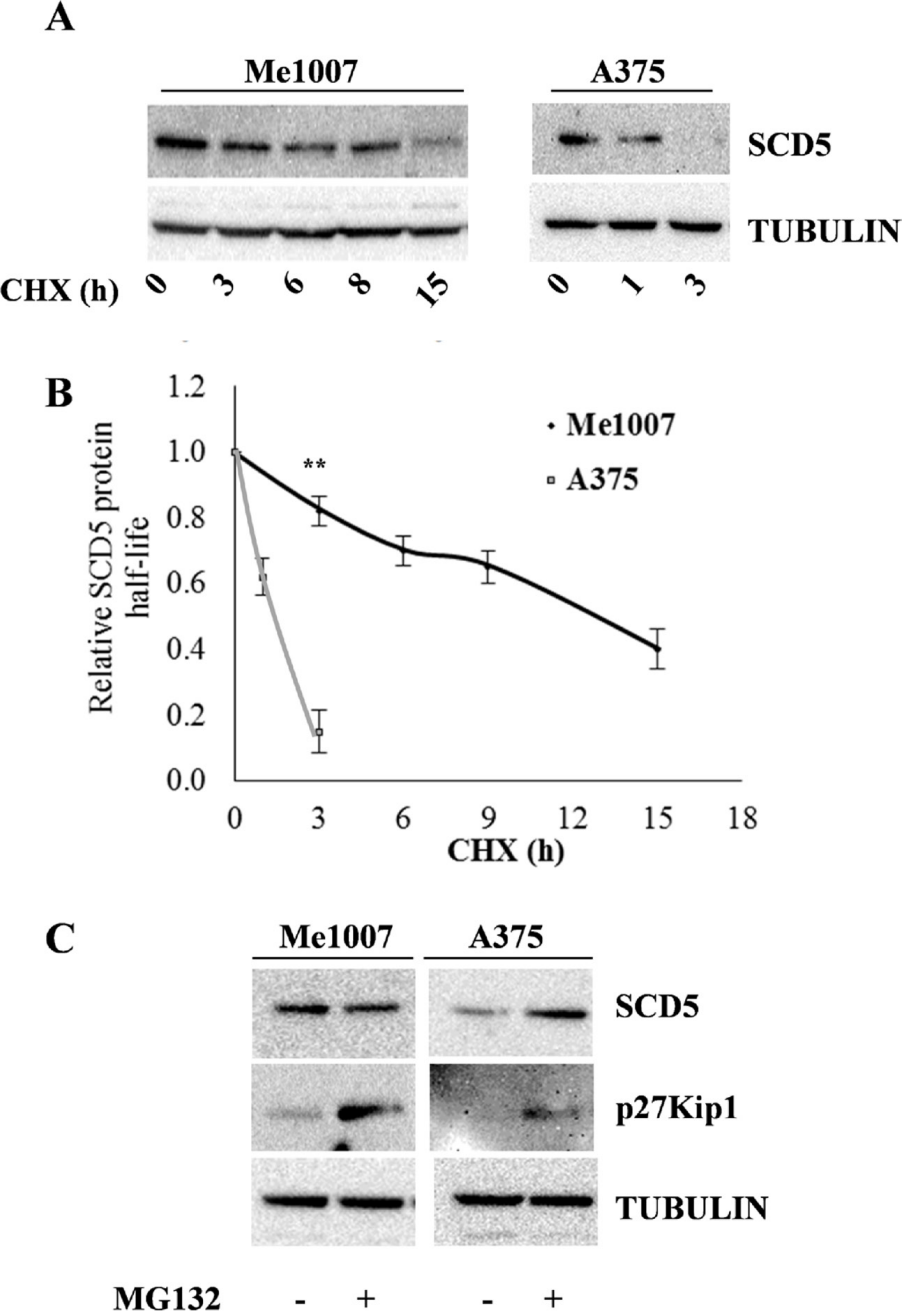
Evaluation of SCD5 protein stability and proteasoma-dependent degradation in melanoma cell lines (**A**) Me1007 early primary and A375 metastatic melanoma cell lines were treated with Cycloheximide (CHX) up to 18 hours and SCD5 stability evaluated by western blot analysis at the indicated time points. (**B**) Densitometric analysis of normalized samples shows the significantl longer half-life of SCD5 protein in Me1007 compared with A375 cell line. (**C**) Western blot analysis shows the MG132-dependent accumulation of SCD5 in A375, but not in Me1007 cells. The cell cycle inhibitor p27Kip1, whose degradation is known to be proteasome dependent, was included as a control.

**Figure 2:**
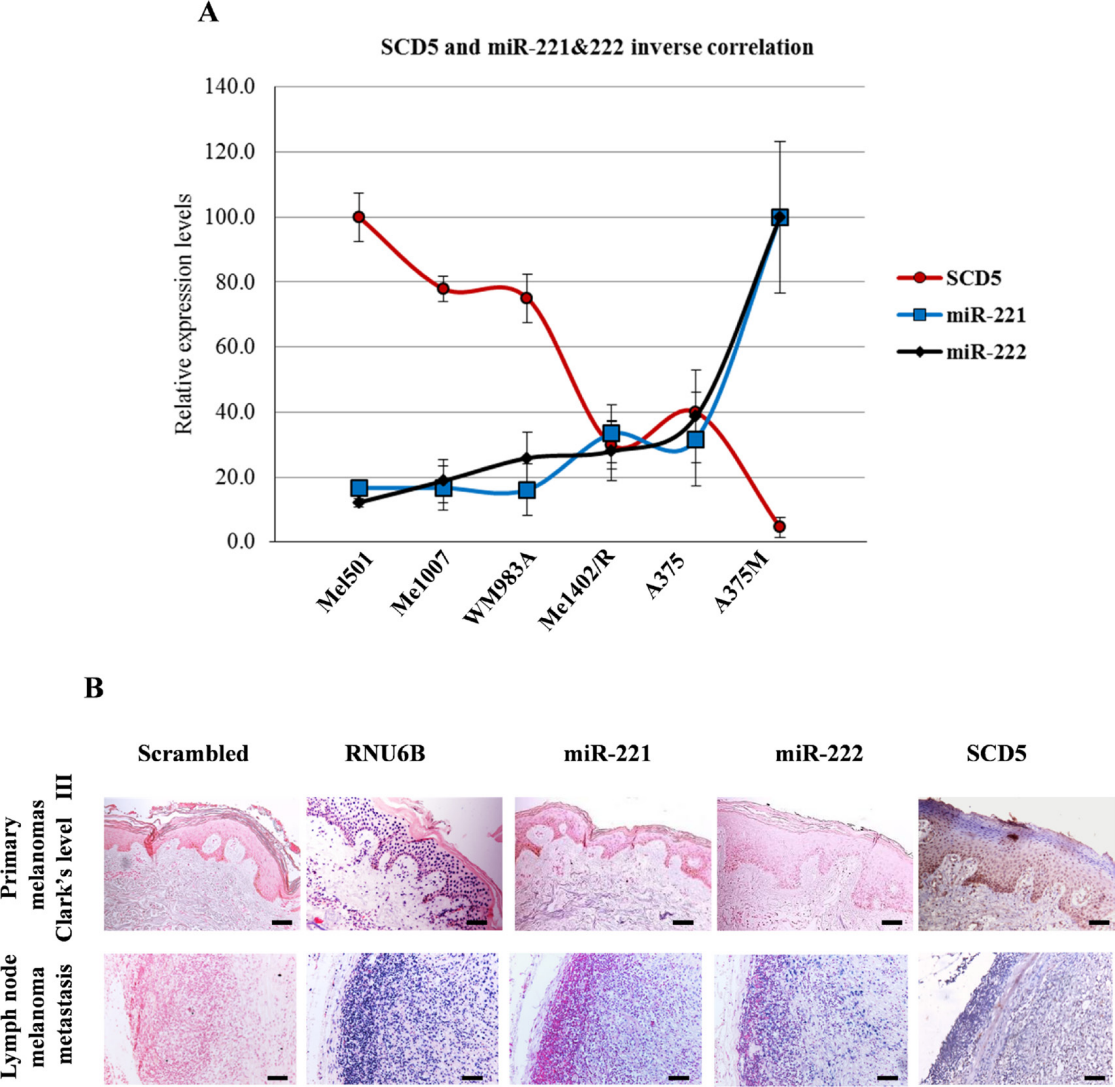
SCD5 and miR-221&222 expressions are inversely correlated in melanoma progression (**A**) The inverse correlations between miRs and their target SCD5 are shown. Relative expression values, reported as arbitrary units, were obtained by qRealtime PCR and densitometric analysis of western blot. (**B**) *In situ* hybridization of miR-221&222 and immunohistochemistry of SCD5 performed on human bioptic specimens. One primary melanoma, Clark’s level III, and one lymph node metastasis are shown as representative. Bar, 100 μm. Scrambled and RNU6B correspond to negative and positive controls of ISH, respectively.

On this basis, we investigated the regulatory mechanisms underlying SCD5 down-regulation associated with melanoma progression. Hence we evaluated the possible involvement of some key epigenetic mechanisms, as DNA methylation and/or histone modifications considering that SCD5 might be included in the number of tumor suppressor genes epigenetically inactivated during tumorigenesis [17]. Actually, utilizing demethylating drugs, as 5-aza-2′-deoxycytidine (5AzaCdR), or drugs that inhibit class I and/or class II histone deacetylases (HDACs), as the pan-inhibitor trichostatin A, we did not obtain significant increases of SCD5 expression levels. In the A375M advanced melanoma, SCD5 appears only slightly unblocked by 5-aza alone whereas no effects at all seemed associated with acetylation. The expected induction of the cyclin-dependent kinase inhibitor p21, included as a positive control, confirmed the effectiveness of the treatments (data not shown).

We then looked for different SCD5 mRNA and/or protein stabilities associated with melanoma progression. The mRNA half-life was evaluated in presence of the mRNA synthesis inhibitor Actinomycin D (ActD), whereas the protein degradation time was assessed by treating with the protein synthesis inhibitor cycloheximide (CHX). For this analysis, we selected the early primary Me1007 and the metastatic A375 cell lines, as representative of initial and advanced stages. Although, the basal amounts of SCD5 mRNA were different between Me1007 and A375, according to the progression stages, being highly expressed in the first and low but detectable in the latter (Supplementary Figure 1A), we did not observe significant differences in their degradation rates (Supplementary Figure 1B). In both cell lines the amount of SCD5 mRNA was approximately halved 15 hours after the ActD treatment. Conversely, the degradation time of SCD5 protein, evaluated in the same cell lines up to 18 hours after CHX action, evidenced significantly accelerated protein degradation of SCD5 in the metastatic A375 cell line (half-life ∼90 min), compared with the slow rate in Me1007 primary melanoma (half-life of approximately 12 hours) (Figure 1A, 1B).

Finally, we examined whether SCD5 regulation would possibly go through proteasome ubiquitination. Notably, treatment with the proteasome inhibitor MG-132 blocked SCD5 degradation in A375 cells, whereas it did not seem to affect the protein level in Me1007 cells (Figure 1C), at least up to 15h, thus further indicating a different regulatory mode of this pathway.

Essentially, we detected a significantly more stable SCD5 protein in primary compared to metastatic melanoma cell lines, in good agreement with the expression pattern detected in our panel of cell lines [9].

### SCD5 is a direct target of miR-221&222

**Figure 3:**
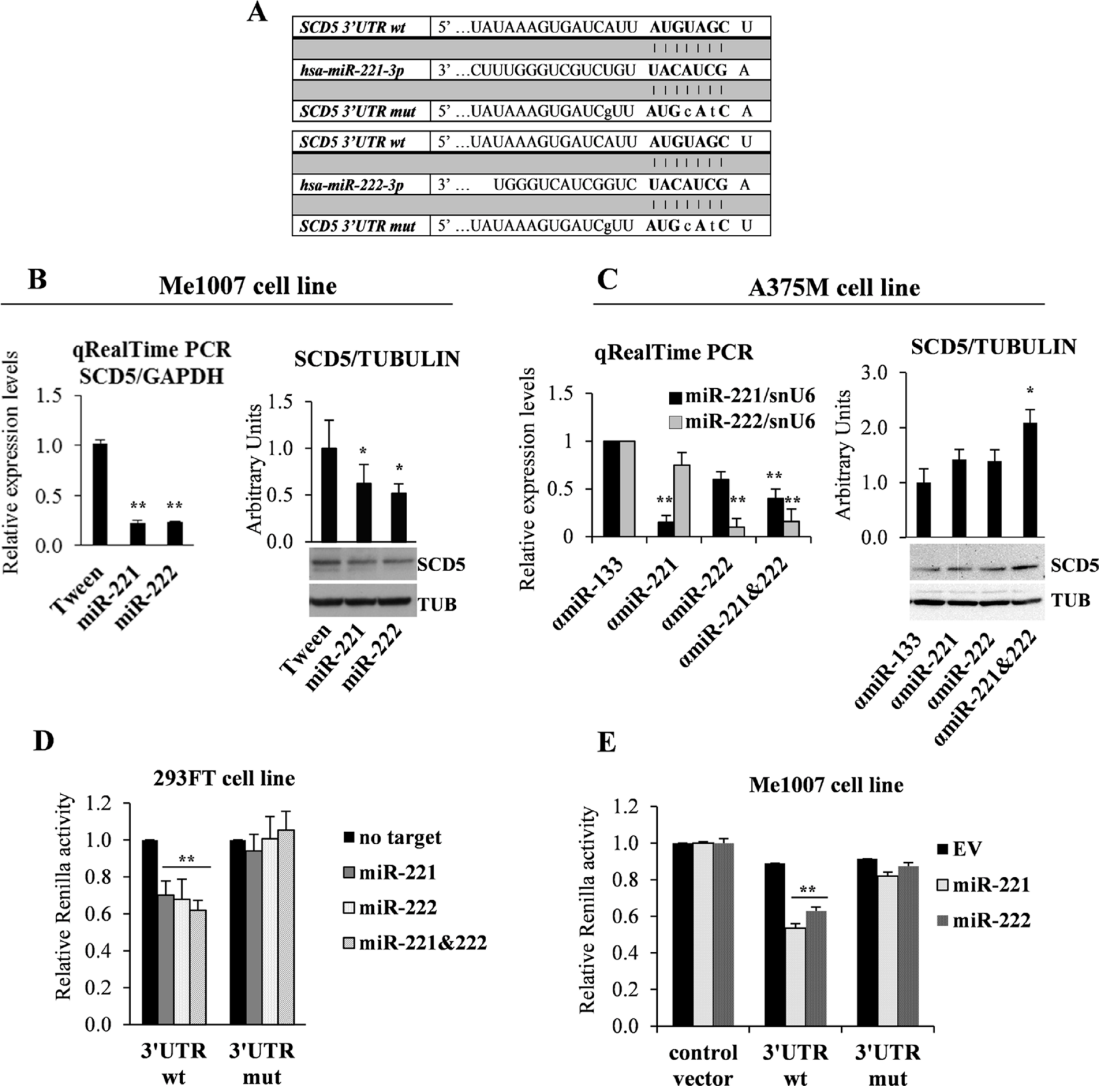
miR-221&222 direct targeting of SCD5 (**A**) Schematic seed pairing between SCD5 3′UTR and hsa-miR-221&222 is shown by bars. The seed sequence is indicated in upper case bold letters, while lower case letters represent mutated nucleotides. (**B**) qRealTime PCR and representative western blot analysis of SCD5 down-regulation in miR-221&222- vs Tween-transduced Me1007 primary melanoma cell line. (**C**) qRealTime PCR of A375M metastatic melanoma transfected with antagomiR-221&222 (αmiR), alone or in combination, confirmed the correct miR down-regulation and the consequent increase of SCD5 expression in co-trasfection condition. Tubulin (TUB) was used as loading control and αmiR-133 as a negative control. Luciferase reporter assays, performed in (**D**) the 293FT transfected cell line and (**E**) Me1007 melanoma lentivirally infected with miR-221 or miR-222. Mutated nucleotides are shown in (**A**). Results were compared with a no targeting oligomer in the first experiment and with empty vector (EV)-transduced cells in the second one. (^*^p < 0.05, ^**^p < 0.01).

Considering the inverse functional correlation between SCD5 and miR-221&222 during melanoma progression [9, 18] and the predicted presence of one conserved binding site for these miRs in the 3′ Untranslated Region (3′UTR) of SCD5 mRNA (http://www.targetscan.com) (Figure 3A), we hypothesized the involvement of these microRNAs (miRs) in SCD5 regulation. According to this hypothesis, SCD5 levels appeared compatible with miR-221&222 targeting evaluated by qRealTime PCR and western blot in melanoma cell lines (Figure 2A) as well as by *in situ* hybridization and immunohistochemistry on primary bioptic samples (Figure 2B). SCD5 was highly expressed in primary tumors, where this couple of miRs was barely or not detectable, whereas the opposite pattern was visible in advanced melanomas (Figure 2A, 2B).

The expression pattern of SCD5 was then analyzed in Me1007 melanoma enforced to lentivirally express miR-221 or miR-222. SCD5 resulted down-regulated at mRNA and protein levels by both miRs (Figure 3B). Accordingly, in antagomiR-treated A375M metastatic melanoma cells, we observed the induction of SCD5 after miR-221 and/or -222 inhibitions (Figure 3C). It is interesting to note the higher effectiveness associated with the abrogation of both miRs on SCD5 re-expression since they share the seed sequence as well as most of their target genes and functional roles.

We then tested whether miR-221&222 were able to directly target SCD5 using a reporter dual luciferase assay. To this end the 3′UTR region of SCD5, containing wild type or mutated miR-221&222 binding sequences (Figure 3A), was cloned downstream to the Renilla open reading frame in a modified psi-Check promoter vector. The initial co-transfection experiments were performed in the 293FT cell line. As shown, transfection of miR-221 and/or miR-222, in presence of SCD5 wild type 3′UTR induced a significant decrement of luciferase activity (roughly 40%) that was higher when transfections were carried out in presence of both miRs (approximately 50%). As a control of specificity, point mutations inserted in the miR binding site restored the Renilla levels (Figure 3D). A similar miR-dependent repression of SCD5 was obtained when the luciferase assays were performed by transfecting the 3′UTR regions of SCD5, either wt or mutated, into the Me1007 melanoma cell line stably overexpressing miR-221 or miR-222. Results, obtained in comparison with empty vector transduced control cells (Figure 3E), confirmed SCD5 as a novel direct target of miR-221&222. Indeed SCD5 down-regulation appears functional to melanoma progression.

### SCD5 increases melanoma differentiation through the up-modulation of MITF transcription factor and the down-modulation of miR-221&222

The microphthalmia-associated transcription factor (MITF) is a master regulator of melanocyte development, differentiation, function and survival [19]. Interestingly, different levels of MITF are reported to exert different effects in melanogenesis, where this protein functions as a rheostat determining the various functional states [20].

**Figure 4:**
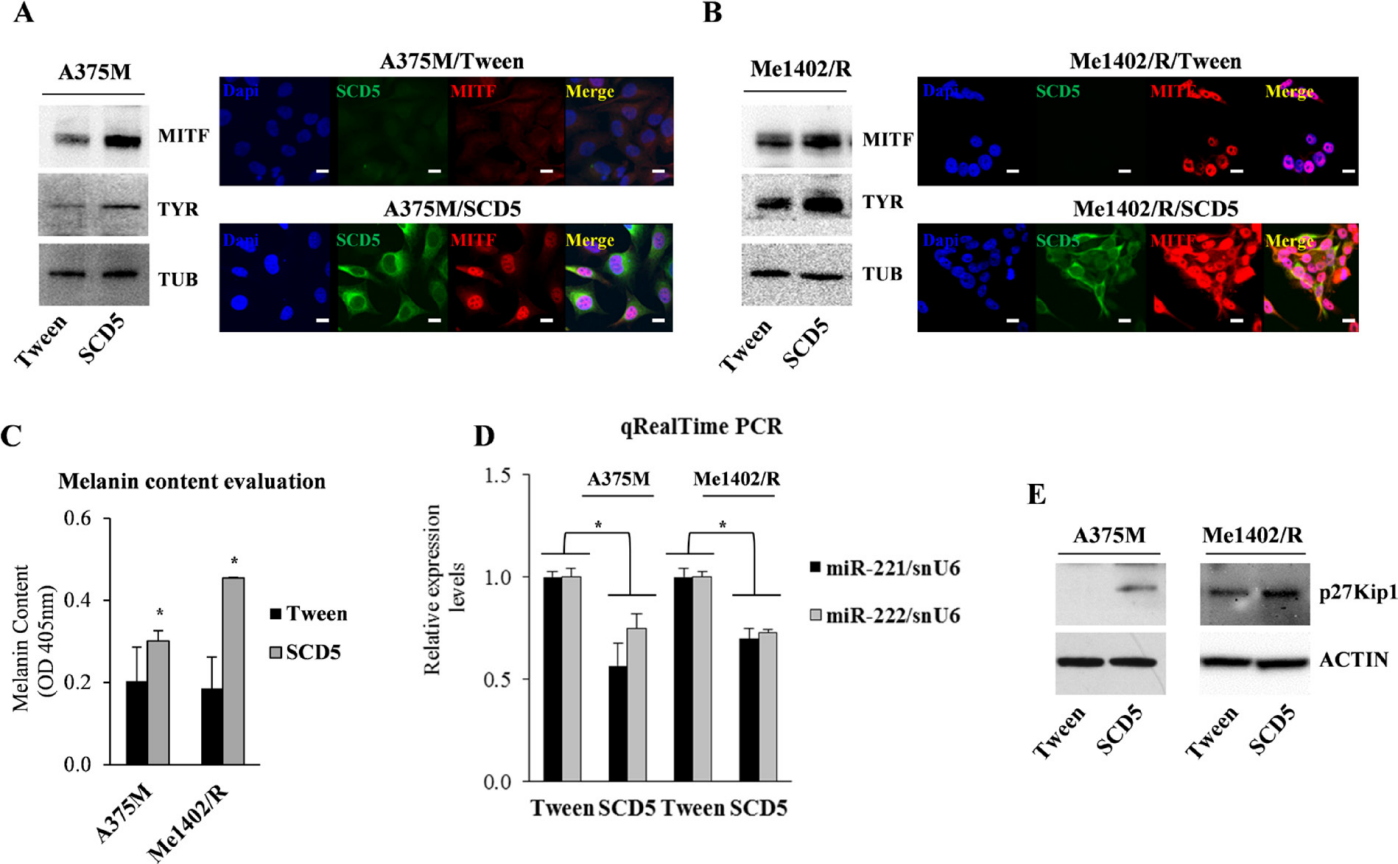
SCD5 overexpression drives melanoma cell lines toward differentiation Western blot and immunofluorescence analyses show a sharp increase of MITF and Tyrosinase (TYR) expression in SCD5-transduced cells compared to Tween controls in (**A**) A375M and (**B**) Me1402/R cell lines. Bar, 10 μm. Tubulin (TUB) was used to normalize. (**C**) Evaluation of melanin content shows its increase in SCD5-trasduced melanoma cell lines compared to the control ones. (**D**) qRealTime PCR show miR-221&222 reduced expression in SCD5 overexpressing melanoma cell lines, associated with (**E**) increased protein level of their target p27Kip1 (^*^p < 0.05).

Actually western blot and IF analyses demonstrated the capability of SCD5 enforced expression to increase MITF, in turn inducing Tyrosinase and increasing the level of melanin in both SCD5-transduced A375M and Me1402/R cell lines (Figure 4A–4C). These data indicate the capability of SCD5 to promote the differentiation program (Figure 4C).

Our previous studies reported MITF down-regulation as a consequence of miR-221&222 targeting of c-KIT and consequently of its downstream pathway, leading to melanogenesis [18]. In addition it was recently reported that MITF represses miR-221&222 promoter and in turn its absence in advanced melanomas contributes to miR-221&222 up-regulation, thus effectively initiating melanoma invasion [21]. Considering the reduced spreading of melanoma cells overexpressing SCD5 [9] and the increased expression of MITF, we evaluated whether miR-221&222 levels were modulated by the presence of SCD5. Indeed, qRealTime PCR confirmed a 40–50% reduction of miR-221&222 expressions in A375M and Me1402/R melanomas overexpressing SCD5 (Figure 4D). The reduced levels of miR-221&222 were correctly associated with increased amount of their target, the cyclin-dependent kinase inhibitor 1B, p27Kip1/CDKN1B (Figure 4E).

All together these results indicate the existence of a negative feedback loop between SCD5 and miR-221&222.

### SCD5 restores sensitivity to ATRA treatment in metastatic melanoma

**Figure 5:**
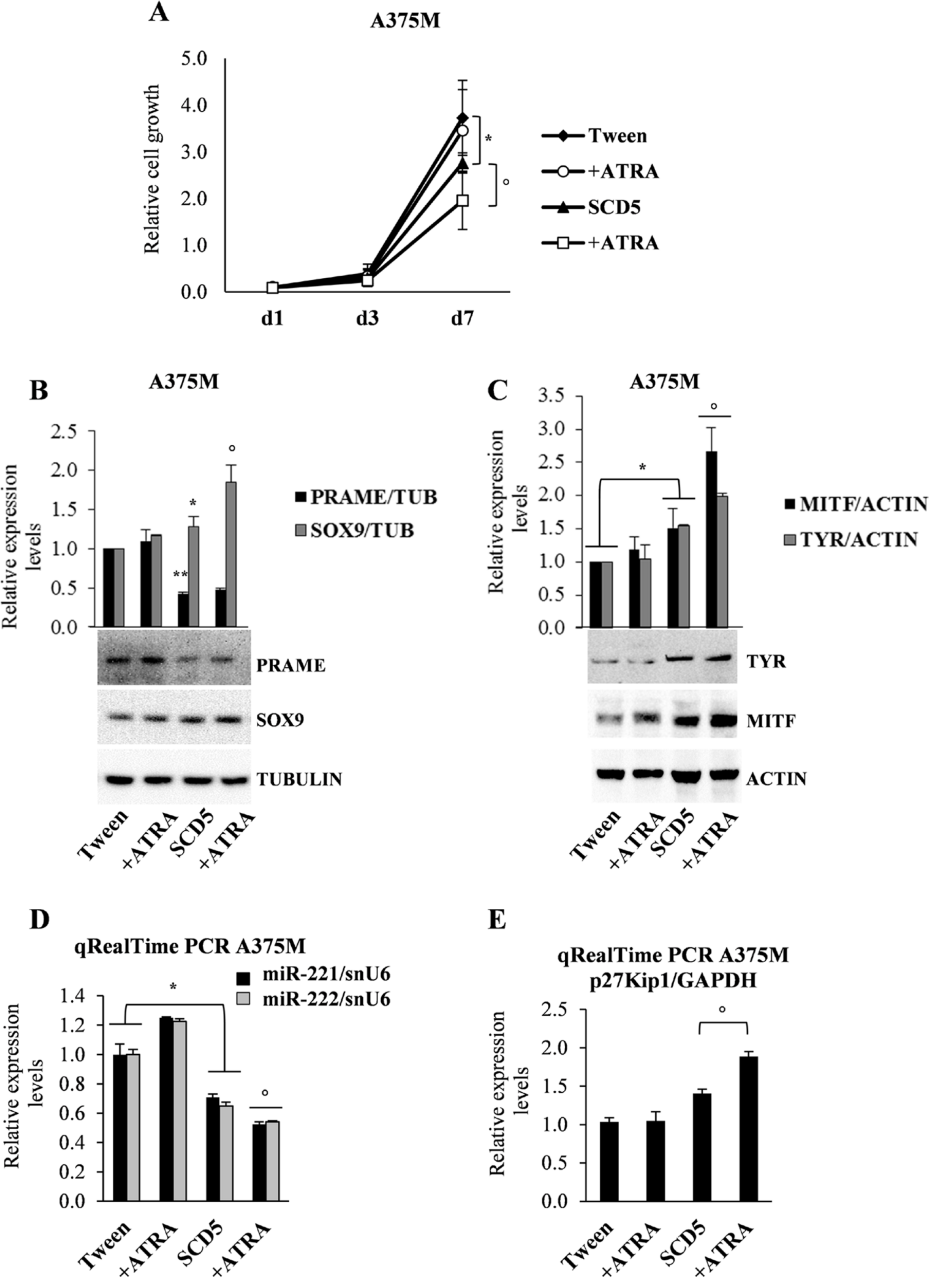
Combined effects of SCD5 overexpression and ATRA supplementation (**A**) Cell growth proliferation evaluated at the indicated time points. Representative WB analysis (bottom) and densitometric quantification (upper) of (**B**) SOX9 and PRAME and (**C**) MITF and Tyrosinase. qRealTime PCR confirmed the ATRA-dependent down-regulation of (**D**) miR-221 and -222 as well as (**E**) up-modulation of their target p27Kip1. (^*^p < 0.05, ^**^p < 0.01 for A375M/SCD5 vs Tween-control); °*p* < 0.05 for A375M/SCD5 plus ATRA exogenous supplementation vs untreated A375M/SCD5).

In several cancers, as leukemia and breast cancer, all trans retinoic acid (ATRA) or its derivatives are successfully utilized, whereas their action is often ineffective in melanoma [22]. Here we evaluated whether SCD5 might enhance melanoma sensitivity to ATRA treatment. To this end, we analyzed the possible functional outcomes on A375M melanoma, known to be ATRA resistant, when transduced either with SCD5 or with the Tween empty vector. These melanoma cells were then compared for their proliferative rates and the expression levels of some possible effectors. Cell growth proliferation of SCD5-transduced A375M cells resulted reduced in comparison to Tween control cells, and was further decreased when cells were co-treated with ATRA. As expected, in view of their resistance, the same treatment had no effect on control cells (Figure 5A). We then evaluated by qRealTime PCR and/or western blot whether SCD5 could modulate the expression level of a number of key factors potentially driving the effects of ATRA supplementation. Actually results showed interest and coherent changes, as we detected the up-regulation of Sex-Determining Region Y-Box 9 Protein (SOX9) which, already induced by SCD5, was further up-regulated by ATRA (Figure 5B). In addition we observed a significant SCD5-dependent down-regulation of Preferentially Expressed Antigen in Melanoma (PRAME) (Figure 5B), a melanoma antigen causally implicated in tumor transformation and acting as a dominant inhibitor of the retinoic acid receptor (RAR) pathway [23]. Looking for the possible effects on melanogenesis, we analyzed the levels of MITF and Tyrosinase, as differentiative factors, and of the cell cycle inhibitor p27Kip1, detecting their increase associated with SCD5 enforced expression and their additional rise due to ATRA treatment (Figure 5C, 5E). Accordingly, the 40% decrease of miR-221&222 consequent to SCD5 overexpression went down to 50% after ATRA treatment (Figure 5D). Thus, the up-regulation of SCD5 expression in human melanomas appear able to restore their sensitivity to ATRA treatment slowing down their cell growth and favoring differentiation.

### SCD5 drives a partial EMT to MET-TF switch in human melanoma

The process of EMT is associated with modulation of transcription factors, surface receptors and secreted molecules, resulting in cytoskeletal reorganization and acquisition of new properties that contribute to tumor progression and metastases formation [24].

In our model the SCD5-associated spreading reduction correlates with the diminished secretion in the tumor microenvironment of SPARC that is known to modify the extracellular matrix (Supplementary Figure 2A, 2B). As a consequence of SPARC reduced secretion into the surrounding microenvironment, SCD5-transduced A375M melanoma and 4T1 mammary carcinoma cells, respectively injected into athymic *Nu/Nu* immunocompromised or immunocompetent syngeneic BALB/c mice were associated with decreased stromal deposition, eventually preventing their metastasization potential [9].

As in melanoma the EMT-like process is influenced by the action of SPARC [11], we evaluated whether SCD5 restored expression might be able to modulate the transcription factors playing a key role in this program.

**Figure 6:**
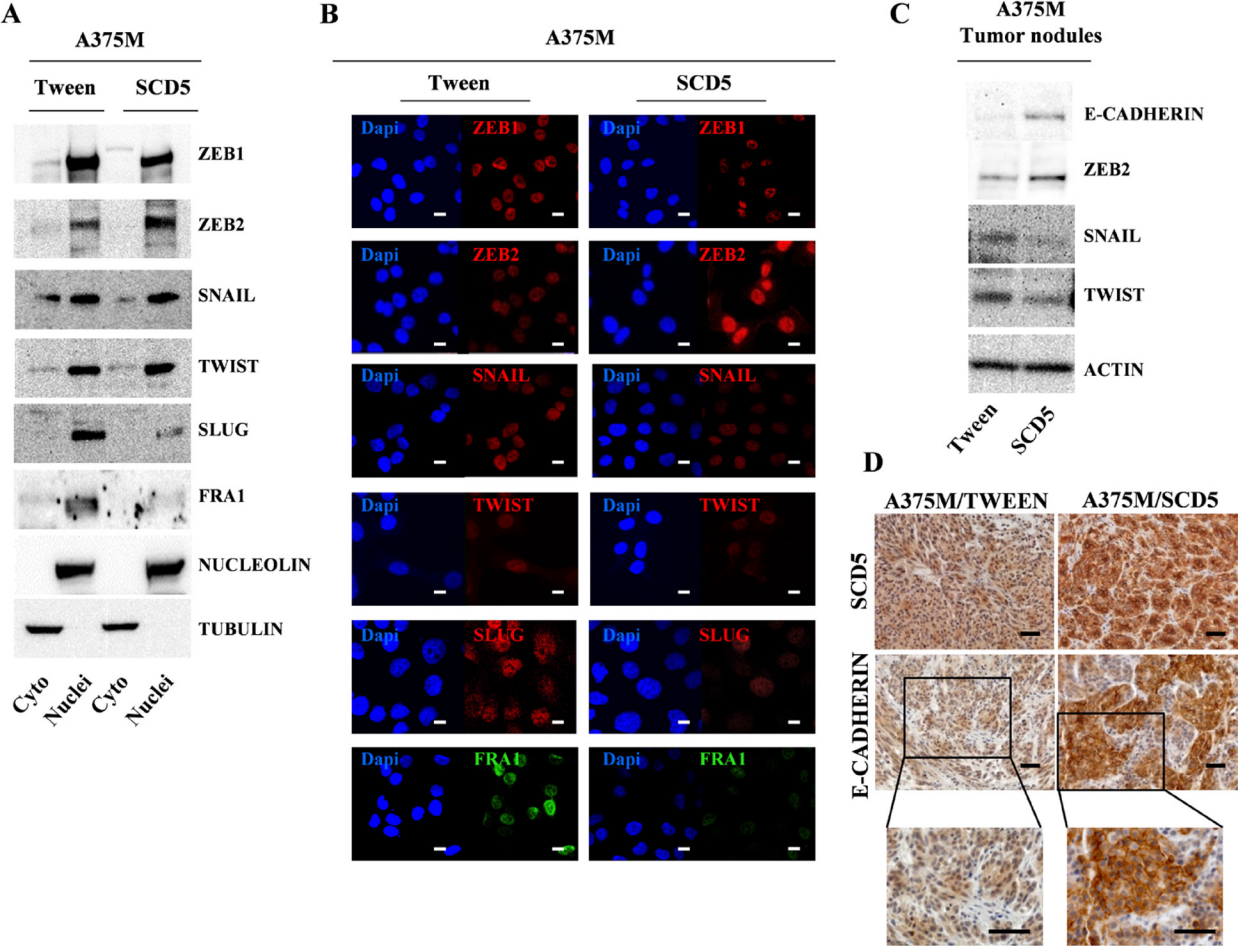
SCD5 overexpression favors a mesenchymal to epithelial transition Evaluation of EMT-TFs levels in purified cytoplasmic and nuclear extracts from A375M/SCD5 vs Tween control cells by (**A**) western blot and (**B**) immunofluorescence. Bar, 10 μm. Tumor nodules derived from Nu/Nu mice s.c. injected with the same cell lines analyzed by (**C**) western blot and (**D**) Immunohistochemistry confirmed the SCD5-dependent regulation of some key EMT-TFs. Bar, 100 μm.

According to the reduced malignancy of SCD5 overexpressing cells, western blot analysis on purified nuclear and cytoplasmic extracts from SCD5-transduced and control cells together with immunofluorescence data showed a significant reduction of Zinc Finger E-Box Binding Homeobox 1 (ZEB1) and Snail Family Transcriptional Repressor 2 (SNAI2/SLUG) paralleled by ZEB2 up-regulation. Also, the FOS Like 1, AP-1 Transcription Factor Subunit (FRA-1), an AP-1 family member whose expression has been associated with melanoma progression, was virtually abrogated (Figure 6A, 6B) [25]. Thus, we checked for the possible induction of E-cadherin, whose functional loss at the cell-cell junctions represents one of the hallmarks of the EMT [26]. We then not only assessed E-cad expression level, but also its localization, both *in vitro* in melanoma cell lines and *in vivo* in tumor nodules recovered from Nu/Nu mice subcutaneous (s.c.) injected either with A375M/SCD5 or A375M/Tween cells. Although no significant increase of E-cadherin amount was detected by IF analysis in A375M/SCD5 cells cultured *in vitro*, we were able to highlight an incomplete relocation of E-cadherin, present as cytoplasmic spots at the external boundary of the cells, differently from the mostly perinuclear distribution observed in control cells (Supplementary Figure 3C top panel). Interestingly, as demonstrated by western blot and IF, these small dots were increased when melanoma cells were treated with 5AzaCdR, according to the methylation-dependent suppression of E-cadherin in advanced melanoma [27]. Once again, despite increased expression, E-cadherin did not appear to be able to reach the cell membrane (Supplementary Figure 3A and 3C bottom panel). Immortalized human keratinocytes (HaCaT) were used as positive controls of E-cadherin expression and cell membrane localization (Supplementary Figure 3B).

More striking results were obtained in the *in vivo* model. In fact western blot analysis of proteins extracted from A375M/SCD5 tumor nodules, in addition to Snail Family Transcriptional Repressor 1 (SNAI1/SNAIL) and Twist Family BHLH Transcription Factor (TWIST) reduction and ZEB2 up-regulation, showed the induction of E-cadherin (Figure 6C). More important, IHC analysis, besides confirming the presence of human melanoma cells highly positive for SCD5, showed the induction of E-cadherin, but also its correct localization at cell membrane levels (Figure 6D).

All together these changes indicate that SCD5 restored expression reduces the metastatic potential of melanoma cells by favoring a Mesenchymal-to-Epithelial transition.

### Effects of oleic acid supplementation

Considering that in melanoma SCD5 plays its main role by converting stearic into oleic acid (OA) and in view of the reported capability of OA to mimic SCD5 functional effects reducing melanoma malignancy [9], we finally evaluated whether the exogenous supplementation of OA could be per se active on EMT-reversion as well as on melanoma differentiation. The A375M/Tween cells were treated with increasing concentrations of OA (20 to 100 µM) up to 3 days and analyzed by western blot. Results showed a significant modulation of some key EMT-TFs, specifically SNAI2/SLUG reduction paralleled by ZEB2 up-regulation (Supplementary Figure 4A). In addition, the induction of MITF and Tyrosinase expression paralleled by miR-221&222 down-modulation, showed the influence of OA also on melanoma differentiation (Supplementary Figure 4A, 4B). All together these results indicate the capability of OA to mimic the effects produced by SCD5 overexpression (Figure 6A).

## DISCUSSION

The treatment of metastatic melanoma still represents a challenging issue. Besides many other factors, fatty acids are known to affect growth response of cancer cells [28]. Specifically, the ratio of stearate to oleate plays a role in controlling bilayer fluidity, in turn influencing membrane functions [29].

Our previous studies showed the antimetastatic role of the desaturating enzyme SCD5 in both human melanoma and murine mammary carcinoma cells [9]. Interestingly, SCD5 enforced expression displayed a preferential action in desaturating stearic more than palmitic acid, thus prevalently inducing oleic acid production. In view of SCD5 decrease associated with melanoma progression, we looked for the mechanisms possibly underlying its regulation. Indeed, a shorter protein half life of SCD5 was evidenced in advanced melanoma compared to primary ones (Figure 1A), supporting the presence of biological processes aimed at preventing accumulation of SCD5 in metastatic cells. Besides this first line regulation, we demonstrated SCD5 to be finely tuned as a novel target of the oncomir-221&222, in agreement with the contribution of this couple of miRs to the invasive potential of melanoma cells through the repression a number of tumor suppressor and negative regulators of cell growth (Figures 2 and 3) [30].

The functional involvement of SCD5 in some circuitries of the oncogenic transformation was confirmed by the induction of key differentiation and antineoplastic genes consequent to its restored expression in advanced melanomas. Indeed, WB or IF analyses performed in both A375M and Me1402/R cell lines enforced to express SCD5, confirmed on one side the rise of MITF and TYR, involved in the melanogenesis program (Figure 4), on the other a partially reversed Epithelial-to-Mesenchymal Transition, further contributing to the antimetastatic action of SCD5 (Figure 6).

Considering SCD5 as part of the differentiation program, we explored the possibility of its role in restoring the sensitivity to differentiating agents, like ATRA, in the highly resistant A375M cell line [31]. This drug, successfully utilized in the treatment of acute promyelocytic leukemia [32], is under exploited in other tumors, including melanoma, due to early development of resistance. According to the lower malignancy associated with SCD5 re-expression, the growth rate of the A375M/SCD5 cells was slightly reduced by the transgene and further down-regulated by ATRA supplementation. Conversely, no significant effects were obtained by ATRA on A375M/Tween control cells (Figure 5). Investigating ATRA response mechanisms, we focused on PRAME, recently reported as a dominant repressor of RAR signaling in AML, and SOX9, a transcription factor significantly down-regulated in melanoma specimens [33, 34]. Indeed, SCD5 overexpression in metastatic melanoma was sufficient to reduce PRAME, in turn activating MITF and SOX9, which were further increased by ATRA (Figure 5B, 5C). The observed antiproliferative effect was supported by the accumulation of p27Kip1 cell cycle inhibitor as a combined product of ATRA treatment and miR-221&222 down-modulation (Figure 5D, 5E). Thus, we can consider SCD5 as an upstream factor able to directly or indirectly regulate proliferation and differentiation in melanoma. Notably, PRAME was also reported to be modulated through the prostaglandin enzymatic pathway starting from the arachidonic acid (AA), a precursor of the proinflammatory cytokines prostaglandin E2 and leukotriene B4, whose metabolism is competitively inhibited by oleic acid (OA) [35]. Interestingly, these data are in good agreement with our results showing the preferential action of SCD5 toward stearic-to-oleic acid conversion as confirmed by the increased desaturation index (18:1/18:0) paralleled by a 5-fold reduction of AA, evaluated by GC/MS ([9] and data not shown).

One of the key antimetastatic roles associated with SCD5 restored expression in melanoma was the strong intracellular retention of SPARC coupled with its impaired secretion (Supplementary Figure 2 and [9]). SPARC overexpression was associated with highly aggressive human melanomas and its secreted fraction underlies the communication between tumor cells and surrounding microenvironment [36]. As SPARC is involved in the transition from epithelial to mesenchymal phenotypes, we have hypothesized the possible contribution of SCD5 in reversing the EMT-like process described in melanoma [37].

In line with Denecker [38], which showed ZEB1 and ZEB2 inverse modulation and the presence of ZEB2 as a positive prognostic factor for melanoma patients, we observed the SCD5-dependent up-regulation of ZEB2 paralleled by reduced amounts of ZEB1 (Figure 6A, 6B). It was reported that ZEB2 regulates MITF levels to control melanocyte differentiation and that Slug and ZEB1 are potent repressors of E-cadherin, in turn enhancing migration and invasion. Although we did not find Twist reduction, ZEB1 down-modulation in parallel to ZEB2 and MITF up-modulation seems to be sufficient to restart melanoma cell differentiation program [39].

It is also important to highlight that the *in vitro* OA supplementation was able to recapitulate the actions of SCD5 in controlling the MET switch and inducing a more differentiated phenotype in A375M/Tween cells (Supplementary Figure 4). This result is in agreement with growing data evidencing that the Mediterranean diet, partly through extra-virgin olive oil and its main component OA, exerts some protection in different form of cancer, including melanoma [40].

Finally, we have to evidence the results obtained *in vivo* in tumor nodules recovered from athymic *Nu*/*Nu* immunocompromised mice s.c. injected, either with SCD5- or empty vector-transduced A375M melanoma cells. Differently from *in vitro* studies displaying only a partial EMT reversion associated with SCD5 (Supplementary Figure 3), tumors overexpressing SCD5 showed a correct modulation of the main EMT-TFs, including E-cadherin induction. IHC, besides confirming the presence of human melanoma cells positive for SCD5, showed evident nests with a clear induction of E-cadherin correctly localized at cell membranes (Figure 6C, 6D).

These data are aligned with the requirement of microenvironmental factors sustaining SPARC-induced EMT, as reported in breast cancer where was demonstrated a functional interplay between myeloid-derived suppressor cells and the extracellular matrix [41].

**Figure 7:**
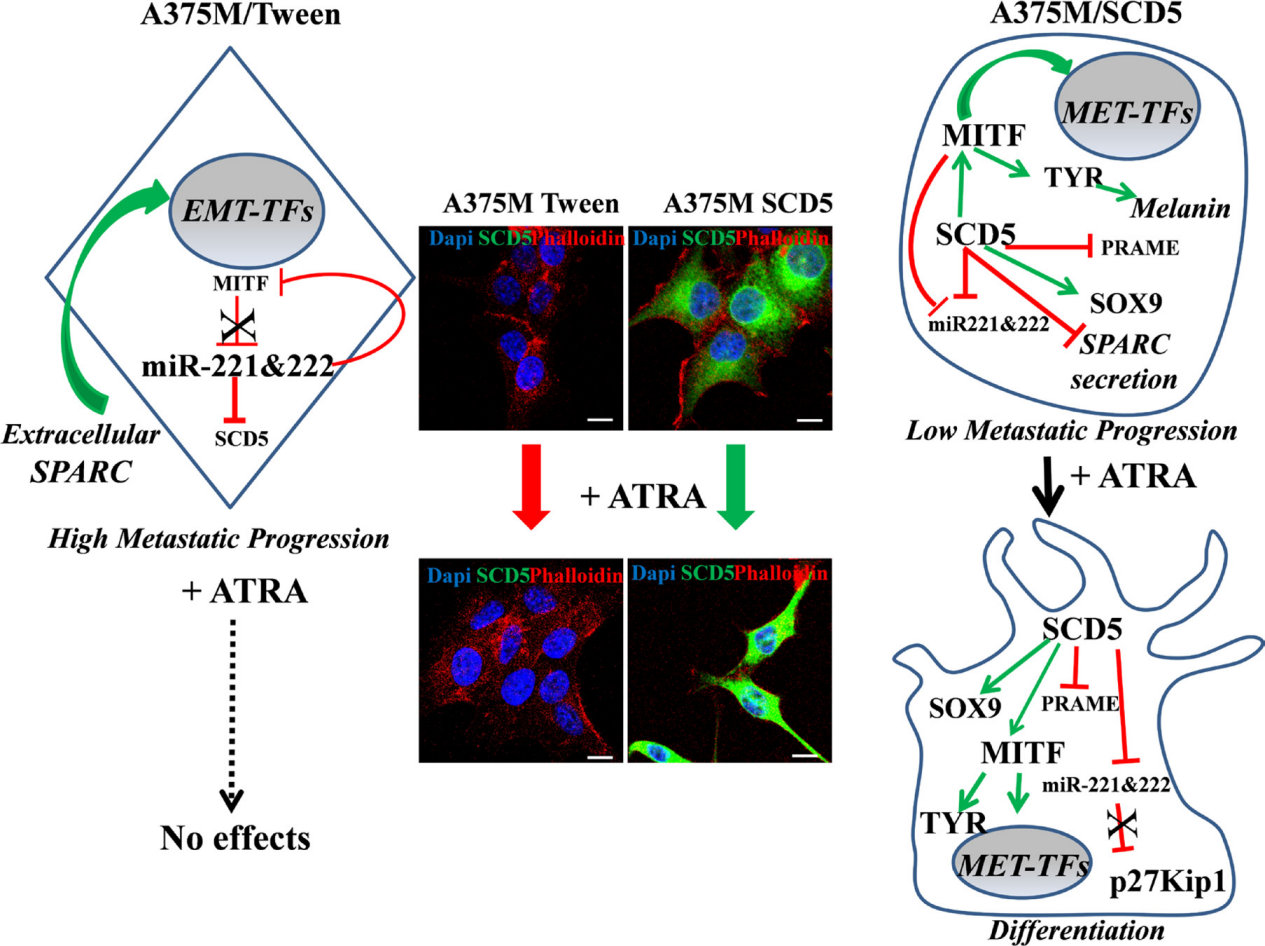
Schematic depicts showing the main SCD5-dependent regulation The effects of ATRA treatment were evaluated on the A375M melanoma, known to be ATRA resistant, transduced either with SCD5 or with the Tween empty vector. This scheme and the corresponding representative IF images show the absence of effect derived by ATRA treatment on A375M control cells and the ATRA-based differentiation of A375M/SCD5 cells, confirming their restored sensitivity to retinoic acid. In addition the key SCD5-dependent molecules are shown. 
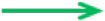
 indicates “induction” and 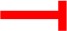 “repression”.

All together these results demonstrate the lack of SCD5 as central to melanoma progression. Indeed, at advanced melanoma stages, as a consequence of SCD5 restored expression we evidenced MITF up-regulation paralleled by miR-221&222 decreases. Hence, in view of the negative cross-regulation between MITF and the oncomir-221&222 [18, 21] and considering the direct targeting of SCD5 by miR-221&222 themselves, we might suggest a self-sustaining circuitry connecting these molecules that, in presence of SCD5, favors the MITF differentiative side, eventually moving the balance from tumor progression toward a less malignant phenotype (Figure 7). Last of all our data indicate the possibility of considering oleic acid or its derivatives for cancer prevention thus including them in the number of adjuvant therapeutic agents.

## MATERIALS AND METHODS

### Cell lines and transduction

Human melanoma cell lines used in the current study (see Supplementary Information Supplementary Table 1) were stabilized from surgical specimens obtained from primary and metastatic tumors (Istituto Nazionale Tumori, Milan-Italy. The A375 cell line was from the American Type Tissue Collection (Rockville, MD, USA) and its metastatic variant A375M was kindly provided by Dr. R. Giavazzi (Istituto Mario Negri, Bergamo, Italy). Normal human epidermal melanocytes from foreskin were obtained from Promocell (Heidelberg, Germany). Melanoma cell lines were authenticated according to standard short tandem repeat (STR)-based genotyping (Biological Bank e Cell Factory, IRCCS San Martino-IST National Institute for Cancer Research Genoa, Italy). The HaCaT cell line, immortalized human keratinocytes used as positive control for E-cadherin expression, was kindly provided by Dr. F. Facchiano (Istituto Superiore di Sanità, Rome-Italy).

Overexpression of SCD5 in Me1402/R and A375M and of miR-221&222 in Me1007 melanoma cell lines was obtained by using a lentiviral vector system, as reported ([9, 18] and Supplementary Figure 5). Chemically modified antisense oligonucleotides (antagomirs) have been used to inhibit miR-221 and -222 expressions *in vitro* (Dharmacon Inc., Lafayette, CO, USA). According to our previously reported data, antagomir-133a was used as a non-targeting negative control [18]. Transfections were performed using Lipofectamine 2000 (Life Technologies, Carlsbad, CA, USA) according to the manufacturer’s instructions.

### Culture conditions

Human melanoma cell lines derived from tumors at different stages of progression have been cultured as described before [9].

The early primary Me1007 and the metastatic A375 cell lines were treated with actinomycin D (2 μg/mL), cychloeximide (CHX, 50 μg/mL) and MG132 (10 μM) (Sigma-Aldrich, Saint Louis, MO, USA) at different time points to evaluate SCD5 mRNA and protein stabilities and proteasome-dependent SCD5 degradation pathway.

A375M cells, transduced either with Tween empty vector or with SCD5, were treated for 72 hours with 5-aza-2′-deoxycytidine (5AzaCdR, 2.5 and 5 μM) (Sigma-Aldrich) for epigenetic analyses. All-trans retinoic acid (Sigma-Aldrich) was used in the same cell lines at 10^–5^/10^–6^ M at day 1, 3 and 7 of culture for proliferation index evaluation (colorimetric assay XTT-based Roche Molecular Biochemicals, Mannheim, Germany) and differentiation marker analyses. Finally, A375M were cultured with oleic acid (SIGMA Aldrich) for 72 h and subsequently processed for molecular analyses. Selected doses of OA, ranging between 20 and 100 μM OA, were tested and then used looking for the effects on EMT and differentiation processes.

### RNA extraction and qRealTime PCR

Total RNA was extracted by using the NucleoSpin miRNA kit according to the manufacturer’s specifications (Macherey-Nagel GmbH & Co. KG. Düren, Germany). qRealTime PCR was performed by the TaqMan Technology (Applied Biosystems, Foster City, CA, USA), using the ΔΔCt method. Commercial ready-to-use primers/probe mixes (Assays on Demand Products, Life Technologies) are listed: miR-221: #000524; miR-222: #000525; SCD5v1 #Hs01125695_m1, p27Kip1 #Hs00153277_m1. MiR-221&222 and SCD5 expression levels were normalized by using snRNA U6 (#001973) and GAPDH (#4326317E) assays, respectively.

### Western blot and immunofluorescence analysis

Western blots were performed according to standard procedures. Total proteins were isolated from cell lysates or from tumor nodules by using NP40 cell lysis buffer and separated by the precast NuPAGE polyacrylamide gel system (Life Technologies Carlsbad, CA, USA). Where indicated, nuclear and cytoplasmic proteins were purified and analyzed following standard protocols. Protein concentration was measured by the Bradford protein assay (Biorad Hercules, CA, USA). The expression levels were quantified using the AlphaView software (ProteinSimple San Josè CA USA).

Immunofluorescence analysis was run according to standard procedures. Briefly, semi-confluent cells were fixed in 4% (w/v) paraformaldehyde (Sigma-Aldrich) and subsequently permeabilized and saturated at room temperature. After incubations with primary and specific fluorophore-conjugated secondary antibodies (Alexa Fluor, Molecular Probes Eugene, OR, USA), slides were mounted with SlowFade anti-fade reagent containing DAPI (Molecular Probes, Eugene, OR, USA). Cellular staining was analyzed by Olympus F1000 laser-scanning confocal microscopy (Olympus, Tokyo, Japan).

### Melanin content evaluation

Melanin content was measured in duplicate at least twice for each cell line as previously described [42]. Briefly, melanoma cells were lysed in NaOH (1N) and the relative melanin content determined by optical density at 402 nm by using fluorometry (Victor X3, Wallac–Perkin-Elmer 2030 software v. 4.00).

### Immunohistochemical and in situ hybridization analyses

For *in vivo* assays, empty vector- or SCD5-transduced A375M cells in exponential growth phase were subcutaneously (s.c.) injected at the dose of 10^6^ cells into adult athymic nude mice, minimum *n* = 5 mice/group (Charles River, Calco, Italy). After evaluation of tumor growth, nodules were isolated for molecular analyses [9]. Specifically, paraffin embedded murine tumor nodules were treated for SCD5 and E-cadherin immunohystochemical studies. Serial sections were subjected to heat-mediated antigenic retrieval (pH 9.0 Tris–EDTA buffer), signals revealed with a polymeric system (Novolink, Max Polymer Detection System, Leica Biosystems, Wetzlar, Germany), and visualized using AECt High Sensitivity Substrate Chromogen Ready-to- Use (Dako Cytomation Liquid AEC Substrate Chromogen System, Agilent Technologies Company, Santa Clara, CA, USA). Concerning, IHC staining of SCD5 on bioptic melanoma, five cutaneous primary (superficial spreading melanoma, Clarks level III) and five lymph nodal metastatic samples were embedded in paraffin, sectioned and pretreated with Sodium Citrate pH 6.0buffer for heat-mediated antigenic retrieval, then combined with a standard ABC technique (Vectastain Rabbit ABC Elite Kit Vector Laboratories INC. Burlingame, CA, USA). Slides were counterstained with hematoxylin and evaluated under a Nikon optical microscope (Nikon Eclipse E1000 equipped with a Nikon DXM 1200 digital camera with dedicated acquisition software (Nikon ACT-1 v. 2.1; Nikon Instruments, Campi Bisenzio, Florence-Italy). Serial sections from the same bioptic human melanoma specimens were subjected to In Situ Hybridization to evaluate miR-221&222 expression levels by using miRCURY LNA microRNA ISH Optimization kit (FFPE) (Exiqon, Vedbaek, Denmark). Specifically we utilized miR-221 (#18115-15), miR-222 (#38499-15) and RNU6B (#699002-340) and Scrambled (#699004-360) sequences, as positive and negative controls, respectively. Specimens were obtained with patient informed consent from the archives of the Human Pathology Section, University of Palermo. Sampling and handling of human tissue material was carried out in accordance with the ethical principles of the Declaration of Helsinki.

### Renilla activity assays

For Renilla reporter experiments, a 551-bp fragment of the SCD5 3′ UTR containing the predicted miR-221 and miR-222 binding sites was amplified by PCR from normal human genomic DNA using a JumpStart™ AccuTaq™ LA DNA Polymerase (Sigma-Aldrich,). The putative SCD5 seed starts at nt 2780 of the SCD5 sequence (NCBI Reference Sequence: NM_001037582.2). The primers utilized were: Forward 5′ GGTGTATAACTCTGACATG 3′ and Reverse 5′ CAGTTTACACATTACCAGTG 3′ for the wild type, Forward 5′ AAG TGA TCg TTA TGcAtC TTC 3′ and Reverse 5′ TCC AGA AGaTgC ATA AcG 3′ for the mutated seed (lower case letters indicate the mutated nucleotides). After sequence analysis, the amplified region was subcloned, either wild type or mutated, in the psiCHECK 2 vector (Promega, Madison, WI, USA) immediately downstream to the stop codon of the Renilla gene. The 293FT and Me1007 cell lines were transfected combining 40 ng of psiCHECK-3′UTR plasmid and 50 pmol of stability-enhanced miR-221 and/or miR-222 oligonucleotides or no targeting RNA control (Dharmacon Inc., Lafayette, CO, USA) with Lipofectamine 2000 (Life Technologies, Carlsbad, CA, USA). The Renilla activity was measured by using the Dual Luciferase assay (Promega Madison, WI, USA) normalized on the Luciferase level. The wt psiCHECK/SCD5 3′UTR cotransfected with the control non targeting oligonucleotide was considered as 100%.

### List of utilized antibodies

SLUG (sc-166476), Tyrosinase (sc-20035), SOX9 (sc-20095), Fra-1 (clone D-3, sc-376148), nucleolin C23 (sc-8031) and ZEB1 (sc-25388) (Santa Cruz Biotechnology, INC, Dallas, TX, USA), Snail (#3895) and p27Kip1 (#2552) (Cell Signaling Technology, Leiden, Netherlands), Microphthalmia (Ab-1, #OP126L, Calbiochem Thermo Fisher, Waltham, MA, USA), Twist (clone 2C1a #ab50887 AbCam, Cambridge, UK), PRAME (TA309818, OriGene, Rockville, MD, USA), ZEB2 (HPA003456, Atlas Antibodies) and SPARC (OSN4.2, #M124 from Takara, Kusatsu, Japan) were used in accordance to the manufacturer’s instructions. A mouse monoclonal and a rabbit monoclonal Abs were utilized against E-Cadherin (clone 36 BD #610181, Transduction Laboratories and clone 24E10, Cell Signaling Technology, Leiden, Netherlands #3195). A specific polyclonal rabbit antibody was generated against a human SCD5 synthetic peptide (aa 313-327) (Eurogentec Group, Liege, Belgium). β-actin (Clone AC-15 #A5441) and α-Tubulin (clone B-5-1-2 #T5168 (Sigma Aldrich St. Louis, MO, USA) were used as loading controls.

### Statistical analysis

Unless indicated otherwise, all data are presented as mean ± standard deviation (SD) and results are representative of at least three independent experiments. Statistical analysis was performed using *t*-test, with *p <* 0.05 deemed statistically significant.

## SUPPLEMENTARY MATERIALS FIGURES AND TABLE


