# Supplementary material for: SCD5 restored expression favors differentiation and epithelial-mesenchymal reversion in advanced melanoma
Source: Oncotarget. 2018 Jan 9;9(7):7567–81. doi: 10.18632/oncotarget.24085 (PMC5800925; doi:10.18632/oncotarget.24085)
Supplement: Supplementary file 1 [file oncotarget-09-7567-s001.pdf]

SCD5 restored expression favors differentiation and epithelial-mesenchymal reversion in advanced melanoma

SUPPLEMENTARY MATERIALS

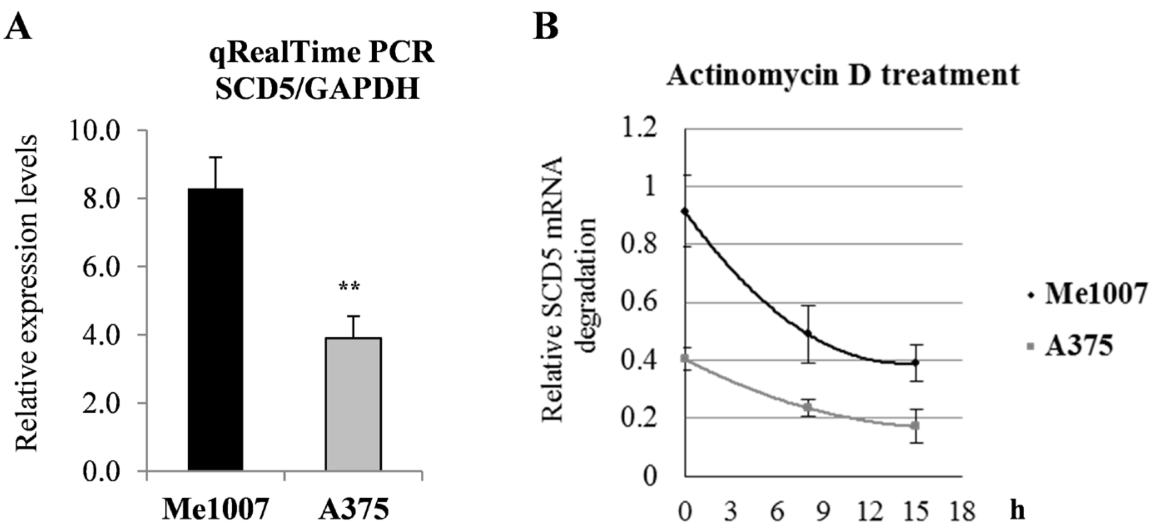

Supplementary Figure 1: SCD5 mRNA stability in melanoma cell lines (**A**) The basal level of SCD5 mRNA was quantitated by qRT-PCR in Me1007 early primary and A375 metastatic melanoma cell lines. (**B**) Evaluation of SCD5 mRNA stability in the same cell lines treated with Actinomycin D up to 15 hours. A representative experiment is shown. Although the initial amounts are quite different, no significant differences were detected in their mRNA degradation rates.

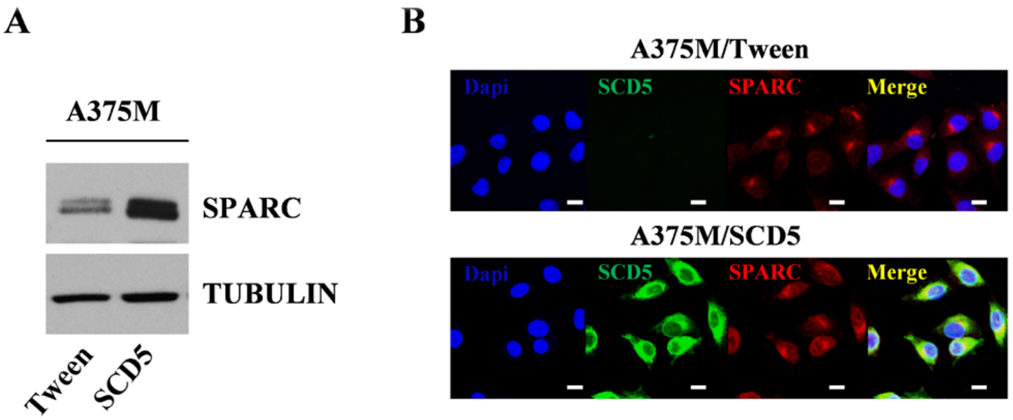

Supplementary Figure 2: SCD5-dependent regulation of SPARC in A375M melanoma. (**A**) WB and (**B**) IF analyses show the intracellular accumulation of SPARC. Bar, 10 µm.

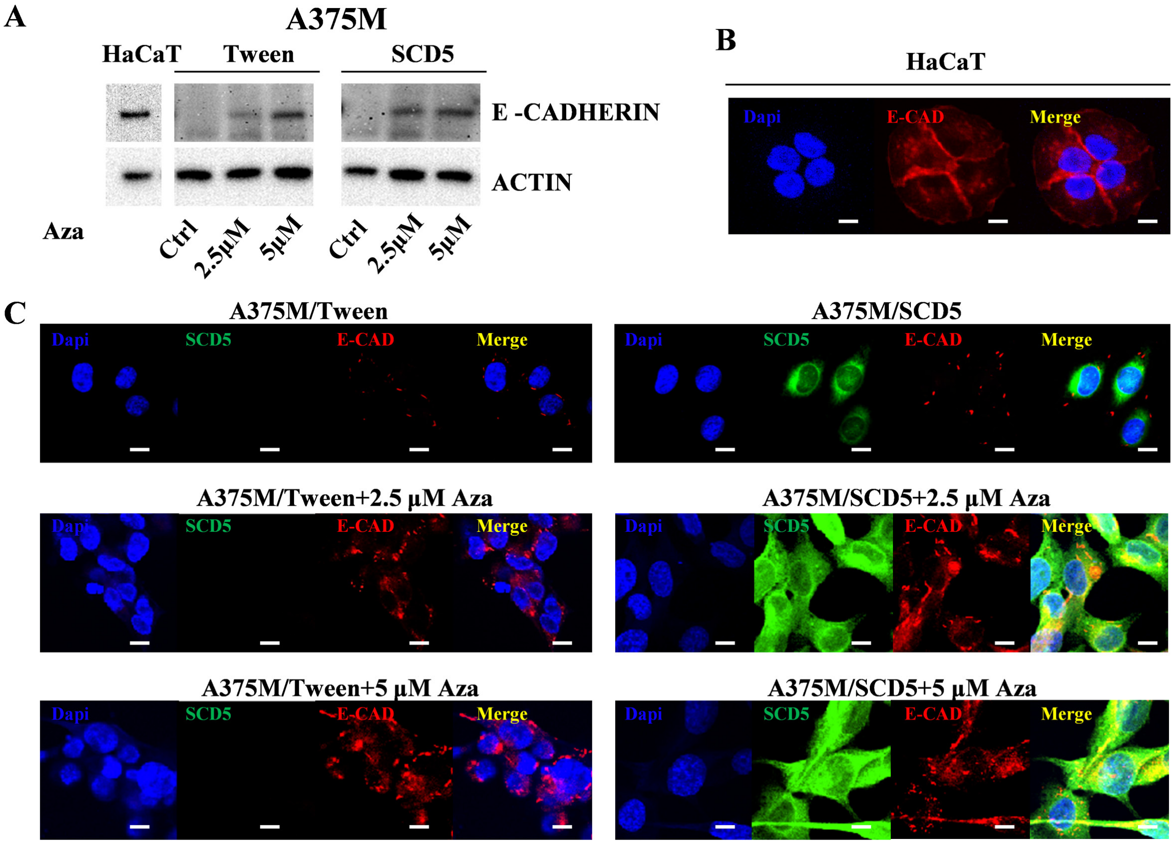

Supplementary Figure 3: E-cadherin epigenetic regulation. (**A**) Representative WB analysis demonstrates 5AzaCdR treatment capability to induce E-cadherin full lenght re-expression in both A375M control and A375M/SCD5 cell lines. HaCaT cell line was used as a positive control. (**B**) IF analysis shows E-cadherin localization at cell membrane junctions in the HaCaT cells and (**C**) at cell periphery as cytoplasmic spots in A375M/SCD5 compared to the perinuclear distribution in Tween control cells. According to E-cadherin demethylation, 5AzaCdR treatment induces E-cadherin expression, particularly in A375M/SCD5 cells. Bar, 10 µm.

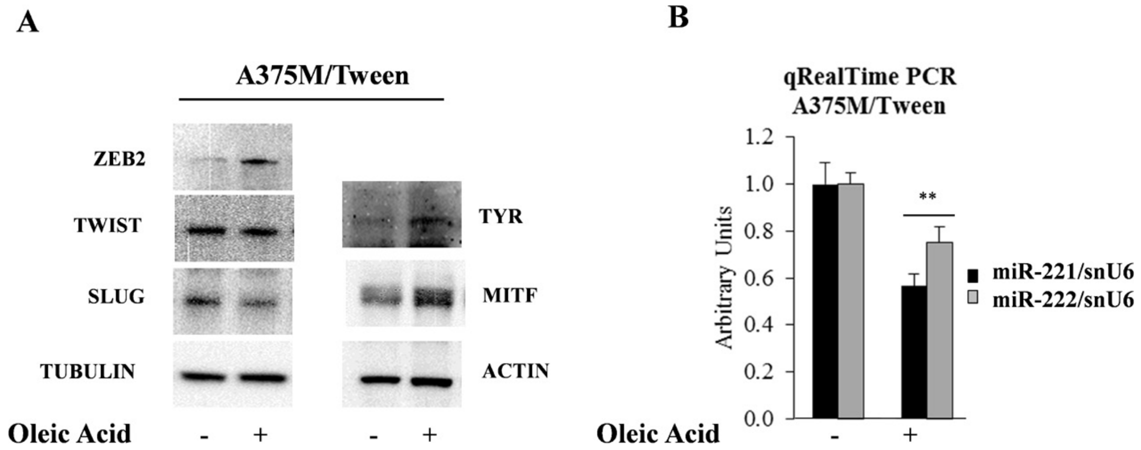

Supplementary Figure 4: Oleic acid reduces the aggressive phenotype of A375M/Tween cell line. (**A**) Western blot evaluation of representative EMT-TFs (left) and differentiation factors (right) in oleic acid (OA) treated A375M/Tween cells, and (**B**) qRealTime PCR showing miR-221&222 reduced expressions.

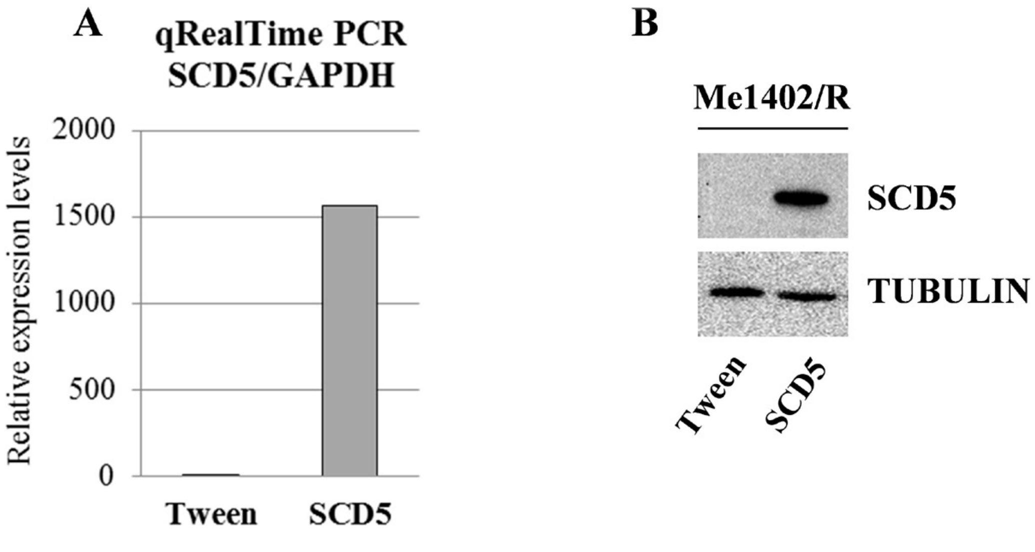

Supplementary Figure 5: SCD5 enforced expression in Me1402/R human melanoma cell line. (**A**) qRealTime PCR and (**B**) western blot analysis comparison between lentiviral transduction of SCD5 and Tween empty vector control.

Supplementary Table 1: Melanoma cell lines analyzed in the current study

Cell line	B-RAF	N-RAS	Type	References
Me1007	wt	wt	Primary Tumor VGP*	1,2
Mel501	wt	wt	Primary Tumor VGP*	3, our unpublished results
WM983A	V600E	wt	Primary Tumor VGP*	4, 3
Me1402/R	V600E	wt	Recurrence of Primary Tumor	1, 2
A375	V600E	wt	Metastatic melanoma	5, 6
A375M	V600E	wt	Metastatic melanoma	7, 8

*VGP vertical growth phase.

REFERENCES

1. Daniotti M, Oggionni M, Ranzani T, Vallacchi V, Campi V, Di Stasi D, Torre GD, Perrone F, Luoni C, Suardi S, Frattini M, Pilotti S, Anichini A, et al. BRAF alterations are associated with complex mutational profiles in malignant melanoma. Oncogene. 2004; 23:5968–77.
2. Colombo MP, Maccalli C, Mattei S, Melani C, Radrizzani M, Parmiani G. Expression of cytokine genes, including IL-6, in human malignant melanoma cell lines. Melanoma Res. 1992; 2:181–9.
3. Supino R, Mapelli E, Sanfilippo O, Silvestro L. Biological and enzymatic features of human melanoma clones with different invasive potential. Melanoma Res. 1992; 2: 377–384.
4. Smalley KS, Lioni M, Dalla Palma M, Xiao M, Desai B, Egyhazi S, Hansson J, Wu H, King AJ, Van Belle P, Elder DE, Flaherty KT, Herlyn M, Nathanson KL. Increased cyclin D1 expression can mediate BRAF inhibitor resistance in BRAF V600E–mutated melanomas. Mol Cancer Ther. 2008; 7: 2876–2883.
5. Tsao H, Goel V, Wu H, Yang G, Haluska FG. Genetic interaction between NRAS and BRAF mutations and PTEN/MMAC1 inactivation in melanoma. J Invest Dermatol. 2004; 122:337–41.
6. Giard DJ, Aaronson SA, Todaro GJ, Arnstein P, Kersey JH, Dosik H, Parks WP. In vitro cultivation of human tumours: establishment of cell lines derived from a series of solid tumours. J Natl Cancer Inst. 1973; 51:1417–1423.
7. Benjamin CL, Melnikova VO, Ananthaswamy HN. Models and mechanisms in malignant melanoma. Mol Carcinog. 2007; 46:671–8.
8. Kozlowski JM, Hart IR, Fidler IJ, Hanna N. A human melanoma line heterogeneous with respect to metastatic capacity in athymic nude mice. J Natl Cancer Inst. 1984; 72: 913–917.
